# Cellulosic Ethanol Production from Weed Biomass Hydrolysate of *Vietnamosasa pusilla*

**DOI:** 10.3390/polym15051103

**Published:** 2023-02-22

**Authors:** Suwanan Wongleang, Duangporn Premjet, Siripong Premjet

**Affiliations:** 1 Department of Biology, Faculty of Science, Naresuan University, Muang, Phitsanulok 65000, Thailand; 2 Center of Excellence in Research for Agricultural Biotechnology, Faculty of Agriculture, Natural Resources and Environment, Naresuan University, Muang, Phitsanulok 65000, Thailand; 3 Department of Agricultural Science, Faculty of Agriculture, Natural Resources and Environment, Naresuan University, Muang, Phitsanulok 65000, Thailand

**Keywords:** weed biomass, *Vietnamosasa pusilla*, phosphoric acid (H_3_PO_4_), glucose recovery, cellulosic ethanol

## Abstract

Lignocellulosic biomass can be used as a renewable and sustainable energy source to help reduce the consequences of global warming. In the new energy age, the bioconversion of lignocellulosic biomass into green and clean energy displays remarkable potential and makes efficient use of waste. Bioethanol is a biofuel that can diminish reliance on fossil fuels while minimizing carbon emissions and increasing energy efficiency. Various lignocellulosic materials and weed biomass species have been selected as potential alternative energy sources. *Vietnamosasa pusilla*, a weed belonging to the Poaceae family, contains more than 40% glucan. However, research on the applications of this material is limited. Thus, here we aimed to achieve maximum fermentable glucose recovery and bioethanol production from weed biomass (*V. pusilla*). To this end, *V. pusilla* feedstocks were treated with varying concentrations of H_3_PO_4_ and then subjected to enzymatic hydrolysis. The results indicated that after pretreatment with different concentrations of H_3_PO_4_, the glucose recovery and digestibility at each concentration were markedly enhanced. Moreover, 87.5% of cellulosic ethanol was obtained from *V. pusilla* biomass hydrolysate medium without detoxification. Overall, our findings reveal that *V. pusilla* biomass can be introduced into sugar-based biorefineries to produce biofuels and other valuable chemicals.

## 1. Introduction

Energy is a vital aspect that determines the socioeconomic development of a nation, especially fossil fuels, which are the principal source of energy. However, combustion of fossil fuels is one of the leading contributors to global warming and other forms of pollution. Mitigating greenhouse gases and diminishing reliance on fossil fuels is the impetus for the pursuit of alternative energy sources. To achieve sustainable development goals, fossil resources should be substituted with biomass feedstocks that can produce various products, including biofuels, green chemicals, and product lines for other industries [1,2,3]. Utilizing lignocellulosic biomass as a sustainable and renewable source of energy could mitigate the consequences of global warming [4]. In the new energy age, the bioconversion of lignocellulosic biomass to green and clean energy has tremendous application potential and makes efficient use of waste [1,2,3]. Among biofuels, bioethanol has been the most researched and manufactured in factories. Bioethanol can reduce carbon emissions, improve energy efficiency, and make a country less dependent on fossil fuels [5,6,7]. Several lignocellulosic materials have recently proven their potential as cellulosic ethanol feedstocks because of their abundance, sustainability, renewable nature, and low cost [1,5,7,8]. Furthermore, several weed biomass species, including *Sida acuta*, *Leucaena leucocephala*, *Achyranthes aspera* [9,10], *Miscanthus × giganteus* [11], *Arundo donax*, *Medicago sativa*, and *Pennisetum giganteum* [12] have been recognized as possible sources of alternative energy because of their high cellulose content. In addition, most weeds in nature survive on marginal lands and produce a high amount of biomass, even though they have limited access to water and nutrition [10].

*Vietnamosasa pusilla*, or Pai Pek (Thai local name), is a weed that belongs to the Poaceae family. It is usually found on plateaus and in semi-deciduous forests in regions with climates that vary seasonally. This weed has the advantage of being able to tolerate drought conditions and grows throughout the year. *V. pusilla* has been declared a “regionally controlled weed”, i.e., it is one of a group of noxious weeds that are risky for a region’s main crop or environment and are likely to spread within that region or to another [13,14,15]. Utilizing this weed’s biomass not only reduces environmental issues but also increases its value. However, research on the applications of this material is limited. One of the most significant obstacles in employing lignocellulosic biomass for biofuel production is the complicated and naturally robust structure of plant cell walls that hinders the hydrolysis of cellulose [16,17,18]. Consequently, several pretreatment procedures have been used to disintegrate the structure of the lignocellulosic biomass, causing the removal of lignin and hemicellulose, which are then transformed into monomer sugars via enzyme hydrolysis. The sugar is then fermented by microbes to produce bioethanol [4,17].

Chemical pretreatment with phosphoric acid (H_3_PO_4_) has been shown to be more effective as several lignocellulosic materials have been successfully pretreated using concentrated H_3_PO_4_ under mild reaction conditions [7]. The hydrolysis efficiency of the pretreated samples was very high, with less inhibitor generation. Furthermore, the remaining pretreated samples did not prevent enzymatic hydrolysis or fermentation [7]. Several studies have demonstrated that H_3_PO_4_ pretreatment of lignocellulosic biomass resources, including *Tripsacum dactyloides*, oil palm empty fruit bunches, and *Populus tremula*, can enhance ethanol yield [7]. In addition, H_3_PO_4_ is less poisonous and corrosive than other acids and is inexpensive compared with other mineral acids. Additionally, phosphoric salts generated by neutralization at the end of the pretreatment process can be utilized as fertilizers, fermentation buffers, and for microorganism nutrition [7,10].

This study aimed to determine the optimal dose of H_3_PO_4_ for pretreating weed biomass (*V. pusilla*) to maximize glucose recovery and bioethanol production.

## 2. Materials and Methods

### 2.1. Material and Preparation

The above-ground *V. pusilla* biomass was collected in March 2022 from the Wang Thong district of Phitsanulok, Thailand (16°51′03.3″ N, 100°44’38.2″ E). The herbarium in Naresuan University’s biology department confirmed the authenticity of the material. The specimen was preserved with record code 05868 for reference purposes at the herbarium.

The samples were rinsed with tap water to remove soil debris and dried under shade for five days. Subsequently, they were cut into long pieces (approximately 5 cm) and crushed into powder using a milling machine (Retsch, Haan, Germany). They were then sieved through a 150–300 µm laboratory test sieve before being placed in bottles at 25 °C for future analysis and experiments.

### 2.2. H_3_PO_4_ Pretreatment

*V. pusilla* Lignocellulosic feedstock was pretreated according to the procedure specified by Premjet et al. [19]. Briefly, “the raw material (3 g) was mixed with different concentrations of H_3_PO_4_ solution (70%, 75%, 80%, and 85% *v*/*v*) in a polypropylene tube at a solid-to-liquid ratio of 1:8. The tube carrying the mixture was then sealed and heated for 60 min at 60 °C in a water bath. The reaction was terminated by adding 25 mL acetone and stirring with a glass rod. The liquid mixture was separated using a fixed-angle centrifuge at 7100× *g* for 15 min at 15 °C. The supernatant was then discarded. This procedure was carried out at least twice, and the solid fraction was rinsed with distilled water until the pH was close to 7.0” [19]. Equations (1) and (2) were applied to calculate the lignin removal (%) and recovery yield (%), respectively [19].

(1) Lignin removal (%)=100− lignin recovery (%)

(2) $$\mathrm{Recovery}\;\mathrm{yield}\;\left(\%\right)=\frac{\mathrm{Solid}\;\mathrm{recovery}\;\mathrm{of}\;\mathrm{each}\;\mathrm{content}\;\left(\%\right)\times \;\mathrm{Treated}\;\mathrm{composition}\;\mathrm{of}\;\mathrm{each}\;\mathrm{content}\;\left(\%\right)}{\mathrm{Untreated}\;\mathrm{composition}\;\mathrm{of}\;\mathrm{each}\;\mathrm{content}\;\left(\%\right)}$$

### 2.3. Chemical Composition Analysis

We followed the technique of compositional analysis outlined by Premjet et al. [19]. The chemical compositions of untreated and treated samples, including monomer sugar, lignin, ash, and extractives, were examined using the National Renewable Energy Laboratory (NREL) development method [19,20,21,22].

### 2.4. Analytical Methods

The details for sugar analysis are provided in a previous report [19]. In brief, “a high-performance liquid chromatography equipment with a refractive index detector was utilized to evaluate the level of carbohydrates. The monomeric sugars were separated employing the Aminex-HPX-87H column with 5 mM sulfuric acid as an eluent at a flow rate of 0.6 mL/min. The RI detector and column temperature were controlled at 55 and 60 °C, respectively. The injection volume was 20 µL” [19].

### 2.5. Enzymatic Saccharification

The saccharification hydrolysis of both the pretreated and raw samples was evaluated using a previously described method [19]. In brief, “at a volume of 10 mL for the mixed reaction, 100 mg of dry biomass, 0.05 M sodium citrate buffer (pH 4.8), and 0.1 mL of 2% sodium azide (*w*/*v*) were added. The enzyme loadings for cellulase (C2730) and β-glucosidase were 30 FPU/g and 60 U/g dry biomass, respectively. The samples were then placed in a shaker incubator for 96 h at 50 °C and 150 rpm. The hydrolysate solution (200 µL) was collected after 12, 24, 48, 72, and 96 h for fermentable sugar analysis. Equations (3) and (4) were used to calculate the hydrolysis efficiency” [19].

(3) $$\mathrm{Glucose}\;\mathrm{digestibility}\;\left(\%\right)=\frac{\mathrm{Glucose}\;\mathrm{released}\;\left(g\right)\times \;0.9}{\mathrm{Glucan}\;\mathrm{in}\;\mathrm{initial}\;\mathrm{biomass}}\times \;100$$

(4) Glucose recovery (%)=[Solid recovery × Glucan content ×1.11× Glucose digestibility]×100

### 2.6. Determination of Biomass Microstructure

The specific method for determination is described in a previous study [10]. In short, “the raw materials and treated samples were freeze-dried. All samples were gold-coated and mounted on aluminum stubs. All coated biomasses were captured employing scanning electron microscope” [10].

### 2.7. Crystallinity of Biomass

The method of analysis was previously explained in a study by Premjet et al. [19]. In short, “both the untreated and treated feedstocks were washed three times with acetone and left at 25 °C for 12 h. They were then ground and sieved, and fraction-size screens ranging from 100 to 150 µm were collected. X-ray diffraction was used to measure the crystallinity index (CrI) of all samples. The samples were scanned between 10° and 40° at 0.2θ(°)/min” [19].

Equation (5) from Segal et al. [23] was applied to estimate the crystallinity index (CrI).

(5) $$\mathrm{CrI}\;=\left(I_{002}-I_{am}\right)/I_{002}\times 100\%$$

where *I*_002_ and *I_am_* correspond to intensity at 2θ = 22.0° and 2θ = 18.0°, respectively.

### 2.8. Preparation of Biomass Hydrolysate (BH) of V. pusilla

The BH medium was prepared using the procedure outlined by Siripong et al. [24]. Briefly, “the BH of *V. pusilla* obtained from enzymatic hydrolysis was boiled for 20 minutes at 100 °C in a water bath to terminate the reaction. The hydrolysate was centrifuged for 2 h at 12,000× *g*. Afterward, it was passed through a glass microfiber filter to eliminate impurities. Then, a rotary evaporator was used to concentrate the hydrolysate until it contained approximately 20 g/L glucose. The hydrolysate was neutralized with 1 M NaOH to increase the pH of hydrolysate to 6. Finally, the BH of *V. pusilla* was maintained at 4 °C for future experiments” [24].

### 2.9. Ethanol Production

This experiment utilized the yeast strain *Saccharomyces cerevisiae* TISTR 5339, acquired from the Thailand Institute of Scientific and Technological Research (TISTR). The BH of *V. pusilla* was fermented with *S. cerevisiae* TISTR 5339 utilizing the methodology explained by Siripong et al. [24]. In Brief, “*S. cerevisiae* TISTR 5339 was grown in 10 mL of liquid Yeast Malt (YM) medium at 30 °C for 18 h at 180 rpm in a rotary shaker incubator to produce a seed culture. *V. pusilla* hydrolysate (50 mL) and a control medium were used to produce bioethanol. Each medium contained 20 g/L glucose (commercial glucose was used as the control medium), 10 g/L peptone, 10 g/L yeast extract, 2 g/L MgSO_4_, and 2 g/L K_2_HPO_4_. The pH of both media was adjusted to 6 with 1 M NaOH, which were aseptically filtered using a 0.02 µm Millipore filter. It was then mixed with 2% seed culture and placed in a shaker incubator at 150 rpm and 30 °C. After 3, 6, 9, 12, 15, 18, 21, and 24 h of incubation, the liquid fraction was harvested for HPLC measurement of sugar uptake and ethanol generation. The optical density (OD) at 600 nm was utilized to monitor the growth of microorganisms using a UV spectrophotometer” [24].

(6) $$\mathrm{Ethanol}\;\mathrm{conversion}\;\left(\%\right)=\frac{\mathrm{Ethanol}\;\mathrm{released}\;\left(g\right)\times \;100}{0.511\;\times \;\mathrm{Glucose}\;\mathrm{consumed}\;\left(g\right)}$$

### 2.10. Statistical Analysis

“Each experiment was performed in triplicate; statistical evaluation of data was performed by analysis of variance using the SPSS version 17.0. The statistically significant difference of each measurement was assessed utilizing Duncan’s test (*p* < 0.05). The result is expressed as the average and standard deviation (± SD)” [19].

## 3. Results

### 3.1. Compositional Analysis

The lignocellulosic *V. pusilla* biomass was used as an alternative biomass source. The chemical composition of the above-ground *V. pusilla* biomass consisted of three primary elements: cellulose, hemicellulose, and lignin (Table 1). The major component of carbohydrates is cellulose (48.1 ± 0.3%), mainly in the form of glucan, followed by hemicellulose composed of xylan (19.2 ± 0.4%) and arabinan (1.2 ± 0.1%). The total amount of carbohydrates was approximately 70%. The lignin content was composed of acid-insoluble lignin (AIL, 23.5 ± 0.1%) and acid-soluble lignin (ASL, 4.4 ± 0.1%). However, the ash and extractive values were 6.1 ± 0.2% and 18.2 ± 0.2%, respectively.

### 3.2. Impact of H_3_PO_4_ on the Chemical Composition of V. pusilla Biomass

To obtain monomer sugar from weed biomass, the *V. pusilla* biomass was treated with 70%, 75%, 80%, and 85% H_3_PO_4_. The major components of this biomass, before and after pretreatment, are listed in Table 2. After pretreatment, the concentration of H_3_PO_4_ had a substantial impact on changes in glucan, xylan, and lignin contents. The xylan content of this biomass decreased by 7.6 ± 0.4%, 4.8 ± 0.3%, and 4.0 ± 0.2% after pretreatment with 70%, 75%, and 80% H_3_PO_4_, respectively. However, the arabinan and xylan content was eliminated by treatment with 70–85% and 85% H_3_PO_4_, respectively (Table 2).

Increasing the H_3_PO_4_ concentration led to a decrease in the AIL, ASL, and total lignin contents (Table 2). Additionally, the total lignin content was reduced from 27.9 ± 0.2% (raw material) to 10.5 ± 0.7%. This accounted for approximately 80% of the total lignin removal. However, the degree of delignification gradually improved to 54.4 ± 1.1%, 68.0 ± 1.2%, 71.2 ± 0.5%, and 82.4 ± 1.2% when treated with 70%, 75%, 80%, and 85% H_3_PO_4_, respectively (Table 2).

Further, the glucan recovery declined steadily to 79.9 ± 0.4%, 78.3 ± 1.2%, 74.7 ± 0.7%, and 68.9 ± 0.7% after pretreatment with 70%, 75%, 80%, and 85% H_3_PO_4_, respectively (Table 2). In contrast, the relative glucan content improved significantly (*p* < 0.05) to 71.4 ± 0.3% and 73.6 ± 1.1% after pretreatment with 70% and 75% H_3_PO_4_, respectively (Table 2). Subsequently, with increases in the concentration of H_3_PO_4_ to 80% and 85%, the relative glucan content decreased to 71.5 ± 0.7% and 70.8 ± 0.8%, respectively; however, this was not a significant difference.

The solid recovery yield gradually decreased with an increase in the concentration of H_3_PO_4_. However, pretreatment with 75% and 80% H_3_PO_4_ did not significantly (*p* < 0.05) affect the solid recovery yields, which were 51.1 ± 0.7% and 50.2 ± 0.4%, respectively (Table 2). The lowest solid recovery yield (46.8 ± 1.7%) was achieved by pretreating with 85% H_3_PO_4_.

### 3.3. Enzymatic Saccharification of V. pusilla Biomass

**Figure 1:**
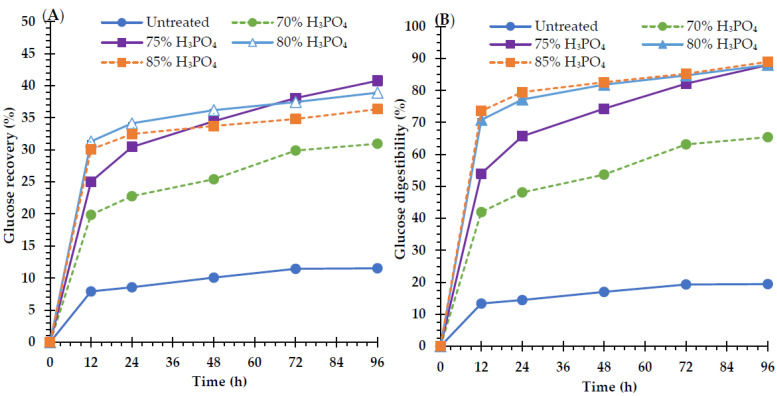
(**A**) Glucose recovery and (**B**) glucose digestibility of untreated and treated *V. pusilla* biomass.

To evaluate the influence of H_3_PO_4_ concentration on glucose recovery and digestibility, cellulose generated after pretreatment with 70%, 75%, 80%, and 85% H_3_PO_4_ was utilized as a substrate for enzymatic hydrolysis, as is shown in Figure 1A,B. After treating raw materials with different concentrations of H_3_PO_4_, the glucose recovery and digestibility yields increased significantly (*p* < 0.05). During enzymatic saccharification, the glucose recovery and digestibility yields improved considerably after 12 h, and then steadily over the next 24 h, 48 h, 72 h, and 96 h. The highest glucose recovery and digestibility yields were observed in both untreated and treated samples after 96 h of incubation. At H_3_PO_4_ concentrations of 70%, 75%, 80%, and 85%, glucose recovery yields were 31.0 ± 0.2%, 40.8 ± 0.3%, 38.9 ± 0.1%, and 36.4 ± 0.1%, respectively (Figure 1A), whereas glucose digestibility yields were 65.4 ± 0.3%, 88.0 ± 0.6%, 88.0 ± 0.1%, and 89.0 ± 0.2%, respectively (Figure 1B). The glucose digestibility yields with 75% H_3_PO_4_ (88.0 ± 0.6%) and 80% H_3_PO_4_ (88.0 ± 0.1%) did not differ significantly, and the greatest glucose digestibility yield (89.0 ± 0.2%) was observed with 85% H_3_PO_4_. Furthermore, this feedstock was pretreated with 70% and 75% H_3_PO_4_, resulting in a substantial increase in glucose recovery yields (31.0 ± 0.2% and 40.8 ± 0.3%, respectively). However, glucose recovery yields declined marginally when the H_3_PO_4_ concentration was increased to 80% and 85%. Moreover, the lowest glucose digestibility yield (19.4 ± 0.0%) and glucose recovery yield (11.5 ± 0.0%) were obtained from the untreated sample (Figure 1A,B). The glucose recovery and digestibility yields were approximately 3.5 and 4.5 times greater, respectively, than those of the raw material. This demonstrates that 75% H_3_PO_4_ is the optimal pretreatment for *V. pusilla* feedstock.

### 3.4. Scanning Electron Microscopy (SEM) Assessment

**Figure 2:**
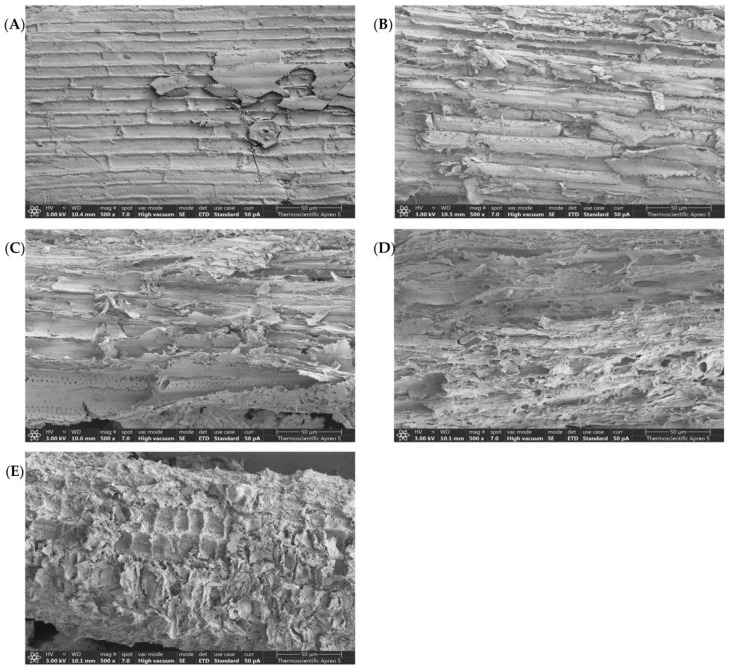
Surface morphology of (**A**) untreated and pretreated samples with (**B**) 70%, (**C**) 75%, (**D**) 80%, and (**E**) 85% H_3_PO_4_.

To gain a more comprehensive understanding of the relationship between biomass morphology and the ability of cellulase to degrade cellulose, the surface morphologies of the untreated and treated *V. pusilla* samples (with varied concentrations of H_3_PO_4_) were evaluated using SEM, as is shown in Figure 2A–E. The SEM images of the untreated samples revealed a highly compressed fibril structure arranged in strict bundles on the surface of the *V. pusilla* biomass (Figure 2A). After pretreatment with 70% and 75% H_3_PO_4_, the surface structure displayed more fractures, peeling, and fiber deconstruction. Consequently, the fibers gradually split until severely disordered, resulting in increased porosity (Figure 2B,C). Surface deconstruction increased with an increase in H_3_PO_4_ concentration. When treated with 80% H_3_PO_4_, the fibrils disintegrated and sustained additional damage (Figure 2D), and pretreatment with 85% H_3_PO_4_ led to collapse of the structure (Figure 2E).

### 3.5. Effects of H_3_PO_4_ on Cellulose Crystalline Structure

**Figure 3:**
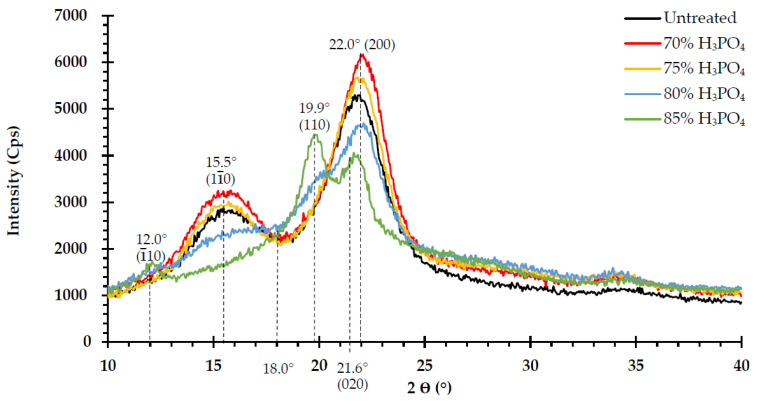
X-ray diffraction pattern of untreated and H_3_PO_4_-pretreated *V. pusilla* samples.

H_3_PO_4_ treatment of the crystalline cellulose of the *V. pusilla* feedstock restricted cellulose digestibility. Therefore, the CrI values of the untreated and treated samples were determined by operating an X-ray diffractometer (XRD). Cellulose crystallinity was altered after treatment with varying H_3_PO_4_ concentrations. The X-ray diffractogram pattern of the untreated sample was identified as cellulose I crystallinity and featured two major peaks at 2θ = 15.5° and 2θ = 22.0° (Figure 3), with a CrI value of 59.5% (Table 3). After the sample was treated with 70% H_3_PO_4_, the highest CrI value (65.0%) was observed. The CrI values were lowered to 63.5%, 49.1%, and 42.9% when the material was treated with 75%, 80%, and 85% H_3_PO_4_, respectively. With 70% and 75% H_3_PO_4_ (Table 3), the XRD patterns were still indicative of cellulose I crystallinity. However, when the sample was treated with 80% H_3_PO_4_, the initial transition from cellulose I to cellulose II was identified because the heights of the two significant peaks of crystalline cellulose diminished more. However, XRD patterns revealed that cellulose I was fully converted into cellulose II crystallinity after pretreatment with 85% H_3_PO_4_ (Figure 3).

### 3.6. Ethanol Fermentation

**Figure 4:**
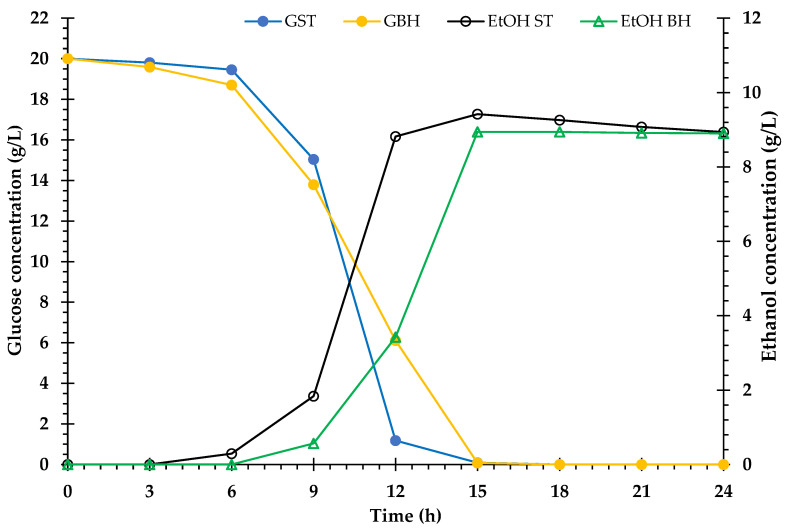
Profiles of glucose consumption and bioethanol fermentation of *S. cerevisiae* TISTR 5339 in standard (ST) medium and biomass hydrolysate (BH) medium. Glucose consumption in standard (GST) medium and glucose consumption in biomass hydrolysate (GBH) medium. Ethanol in standard (EtOH ST) medium and biomass hydrolysate (EtOH BH) medium.

**Figure 5:**
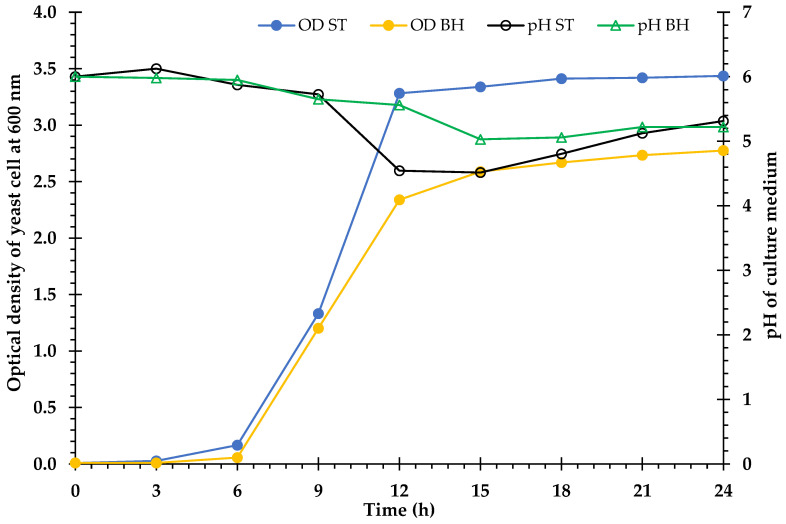
Cell growth of *S. cerevisiae* TISTR 5339 in standard (OD ST) medium and biomass hydrolysate (OD BH) medium. The pH in standard (pH ST) medium and biomass hydrolysate (pH BH) medium.

The BH medium was produced by enzymatic hydrolysis of the *V. pusilla* biomass after pretreatment with 75% H_3_PO_4_. In this experiment, a non-detoxified BH medium containing 20.0 g/L glucose was employed as the culture medium for the production of cellulosic ethanol by *S. cerevisiae* TISTR 5339. The control group consisted of a standard (ST) medium with 20.0 g/L D-glucose (reagent grade, 98%). The patterns of bioethanol formation, yeast cell growth, and glucose consumption in both media are shown in Figure 4 and Figure 5. According to the results, glucose consumption in both the ST and BH media gradually decreased, and the glucose was completely consumed after 15 h of incubation. Ethanol production was observed in the ST and BH media after 6 h and 9 h of incubation, respectively. Subsequently, the ethanol yields in both media steadily increased. The maximum ethanol yields of 9.4 g/L (92.1%) and 8.9 g/L (87.5%) were obtained from the ST and BH media, respectively, after 15 h of incubation. After this stage, ethanol production by the yeast in both media did not increase. However, ethanol production in the BH medium was lower than that in the ST medium (Figure 4). Furthermore, the yeast growth rate profiles in both media were comparable. The maximum growth rate was observed in both media after 12 h of incubation. However, the growth rate of the yeast in the ST medium was greater than that in the BH medium (Figure 5).

## 4. Discussion

### 4.1. Compositional Analysis

According to our results, *V. pusilla biomass* has a higher glucan content than that of various lignocellulosic materials promoted for bioethanol manufacturing, such as spruce (47.1%), pine (45.6%) [25], barley husk (45.7%) [26], Douglas fir (46.1%) [27], corn cob (45.0%) [28], bamboo (44.6%) [29], sugarcane bagasse (43.7%) [30], wheat straw (42.5%) [31], cotton stalk (42.3%) [32], rice straw (41.9%) [33], and eucalyptus (41.4%) [34]. In addition, the glucan content was higher than that of weed lignocellulosic biomass, including that of *Sida acuta* (46.9%), *Achyranthes aspera* (45.9%) [10], *Prosopis juliflora* (45.5%), *Lantana camara* (45.1%), *Saccharum spontaneum* (45.1%), and Siam weed (41.0%) [1]. Regarding the amount of xylan content, it was greater than that observed in sponge gourd fibers (17.4%), banana waste (14.8%) [8], cotton (16.0%), acacia (13.0%) [35], and Japanese cedar (13.8%) [36]. In addition, the lignin content of the *V. pusilla* was equivalent to that of rubber wood (27.6%) [37], but lower than that of several lignocellulosic biomasses promoted for biofuel production, such as oak (35.4%), Japanese cedar (33.5%) [36], switchgrass (31.2%) [38], poplar (29.1%), and hemlock (28.5%) [36]. These results indicate that the lignocellulosic *V. pusilla* biomass has high potential for application in sugar platform-based biorefineries for the manufacture of cellulosic ethanol as well as other importance compounds.

### 4.2. Impact of H_3_PO_4_ on the Chemical Composition of V. pusilla Biomass

The exploitation of lignocellulosic biomass in sugar-based biorefineries requires a pretreatment procedure to break down its recalcitrant structure [19,39,40]. It has been observed that acid pretreatment with H_3_PO_4_ is an extremely successful method for breaking glycosidic links in lignocellulose and dissolving hemicellulose, cellulose, and a small quantity of lignin [3,41,42]. The results indicated that H_3_PO_4_ affected both the partial and entire reduction of xylan from the biomass. Under acidic conditions, hemicellulose is more sensitive to solubility than lignin and cellulose because it is amorphous, has a low degree of polymerization, and is predominantly present in the form of xylan [7,16,43,44]. In contrast, lignin is an amorphous, water-insoluble heteropolymer composed of three cinnamyl alcohol precursors (sinapyl alcohol, coniferyl alcohol, and p-coumaryl alcohol) connected via various links and packed into a lignin layer in the plant cell wall, resulting in resistance to chemical pretreatment [45,46,47]. Consequently, even pretreatment of this biomass with the maximum concentration of H_3_PO_4_ (85%) resulted in only the partial removal of lignin. Nevertheless, both partial xylan and lignin removal led to an increase in relative glucan content in the solid recovery after pretreatment of this biomass with 70–80% H_3_PO_4_ [19,48]. However, pretreatment of feedstock with 85% H_3_PO_4_ led to the breakdown of the linkages of the lignin–carbohydrate complex and the dissolution of the cellulose fibrils and hemicellulose due to the destruction of the hydrogen bonds between the sugar chains, leading to a substantial reduction in solid recovery, glucan recovery, and relative glucan content, and the elimination of xylan content [7,49]. Furthermore, similar effects of H_3_PO_4_ have been observed in various feedstocks, including *Luffa cylindrica* [50], *A. aspera*, *S. acuta* [10], *Hibiscus sabdariffa* [51], *Durio zibethinus* [48], and *Hibiscus cannabinus* [19].

### 4.3. Enzymatic Saccharification of V. pusilla Biomass

The primary goal of lignocellulosic biomass pretreatment is the elimination of hemicellulose and lignin, leaving cellulose in the solid phase for subsequent enzymatic hydrolysis and fermentation [3]. When raw *V. pusilla* biomass was pretreated with varying concentrations of H_3_PO_4_, the glucose recovery and digestibility yields were considerably enhanced for each pretreated sample, depending on the concentration of H_3_PO_4_. These results demonstrate that the effect of H_3_PO_4_ pretreatment on the hydrolysis of each pretreated sample in relation to glucose was more pronounced. In contrast, enzyme hydrolysis of the raw material resulted in the lowest yields for glucose recovery and digestibility. This is because of the stiffness and obstinacy of lignocellulosic feedstock, which inhibits enzyme hydrolysis [1,16,40,44].

During the conversion processes, the lignin in lignocellulosic biomass functions as an inhibitor by preventing enzymes from accessing cellulose and inhibiting enzymatic activity through nonproductive binding. Consequently, eliminating the lignin from lignocellulosic feedstock via acidic pretreatment is crucial in order to minimize chemical and physical barriers and enhance the access of cellulolytic enzymes to cellulose [7,47]. These results demonstrate that increasing the concentration of H_3_PO_4_ positively affects both glucose recovery and digestibility yields, which coincides with the elimination of AIL and ASL. The greatest glucose recovery and digestibility yields were obtained when the feedstock was pretreated with 75% H_3_PO_4_, whereas AIL (66.9 ± 0.2) and ASL (69.4 ± 0.2) were excluded. These quantities represent the total amount of lignin (68.0 ± 1.2%) extracted from the treated biomass (Table 2). These results reveal that the increase in the glucose recovery and digestibility yields resulted from partial delignification due to H_3_PO_4_ pretreatment. In addition, delignification often causes the breakdown of the polymeric network, leading to increased porosity and diminished enzyme inhibitory action [7,19,52]. According to reports, pretreatment of various lignocellulosic biomasses using particularly aggressive methods, such as harsh bases or acids, accelerates the delignification of the feedstock and the disintegration of carbohydrates [53]. This suggests that complete lignin elimination is not required.

The hemicelluloses in lignocellulosic biomasses are physical barriers surrounding cellulose, limiting hydrolysis reactions, restricting enzyme access to cellulose, and lowering cellulase activity. However, lignin has a greater effect than hemicellulose. Therefore, removing the hemicellulose present in the xylan content may accelerate cellulose hydrolysis by expanding the exposure of cellulase to cellulose [1,16,40,44], which is comparable to lignin. In this operation, hemicellulose was effectively eliminated from this feedstock, which was treated with 85% H_3_PO_4_. The glucose digestibility yields of the samples pretreated with 75% and 80% H_3_PO_4_ were not significantly different. The samples treated with 75% H_3_PO_4_ resulted in the highest glucose recovery rates (40.8 ± 0.3%), whereas those treated with 85% H_3_PO_4_ resulted in the highest glucose digestibility rates (89.0 ± 0.2%). Although the glucose digestibility reaches its maximum under certain circumstances, such as at a concentration of 85% H_3_PO_4_ in this study, the total glucose recovery may be lower due to high solid loss (53.2 ± 1.7% in this study) under severe conditions [24,54]. Consequently, the entire glucose recovery process defines the pretreatment parameters used. This study confirmed that pretreatment of *V. pusilla* biomass with 75% H_3_PO_4_ was optimal. At this concentration, approximately 78.3 ± 1.2% and 51.1 ± 0.7% of glucan and solid, respectively, were recovered. This demonstrates that more than 50% of the glucan in the treated biomass was still present. According to reports, the ideal pretreatment condition is one in which the glucose recovery level is the greatest and closest to the initial glucan content of the biomass [48,54]. The increase in both glucose recovery and digestibility yields may be attributed to the disintegration and dissolution of the hemicellulose and lignin, which renders the cellulose present in the treated *V. pusilla* biomass vulnerable to cellulases. In comparison to other lignocellulosic biomasses, the glucose digestibility yield of the feedstock was higher than that of vegetable hummingbird (86.0%), alamo switchgrass (85.0%), eastern gamagrass (80.5%), switchgrass (73.5%) (as reported by Satari et al. [7]), Devil’s horsewhip (86.2%) [10], common wireweed (82.2%) [10], pine (80.0%), and Douglas fir (79.0%) [55]. The glucose recovery was also greater than that of chanee durian peel (38.5%) [48], Douglas fir (31.2%), and pine (28.0%) [55].

### 4.4. SEM Assessment

To accelerate the enzymatic conversion of lignocellulose to monomeric sugar, the innate stiffness of plant cell walls must be disassembled by processing using acids or bases to improve the access of enzymes to the cellulose surface [1,39,47]. The raw material exhibited the structural obstinacy of *V. pusilla*, which is predominantly composed of a lignin–carbohydrate matrix, rendering it extremely resistant to enzymatic hydrolysis [16,40]. The structural transformation of the treated sample was accompanied by a substantial alteration in its chemical composition upon treatment with 70% and 75% H_3_PO_4_. This was because H_3_PO_4_ pretreatment enabled the breakdown of the resistant lignocellulose structure by destroying the hemicellulose and lignin networks and increasing cellulose accessibility. Consequently, modifications in the internal cell wall composition, such as destruction, increased porosity, and loss of recalcitrant structure, increased enzyme accessibility to cellulose [7,11]. These outcomes are consistent with the steady increase in the glucose recovery and digestibility yields and contribute to the maximum glucose recovery and digestibility generated by 75% H_3_PO_4_ pretreatment. Furthermore, the SEM micrographs of both the treated samples revealed a greater extent of degradation of the morphological surface when H_3_PO_4_ concentration was increased to 80% and 85%. This effect is caused by an upsurge in cellulose degradation under harsh conditions, resulting in a considerable decrease in glucose recovery yields [7,11,56,57,58,59,60]. In a previous study, we found that eliminating lignin and hemicellulose, which are physical barriers surrounding cellulose, could be a key factor contributing to the improvement of glucose digestibility and recovery yields [19]. These findings validate the outcome of the enzymatic hydrolysis.

### 4.5. Effects of H_3_PO_4_ on Cellulose Crystalline Structure

Cellulose is a linear polysaccharide composed of highly organized repeating glucose units. These glucan chains are linked by hydrogen bonds between the molecules, resulting in a partly crystalline structure [61,62,63]. Biomass crystallinity is a critical determinant of the enzymatic hydrolysis of cellulose [61,64,65]. In this study, the CrI value of the untreated biomass (59.5%) was lower than those observed for wheat straw (69.6%) and hemp fiber (77.0%) raw materials [62]. The pretreatment of various lignocellulosic biomasses with acids, alkalis, or other processes results in the partial elimination of amorphous components (hemicellulose and lignin) in the biomass, leading to a decrease in the noncrystalline background revealed by XRD and an increase in the CrI value [11,19,47,49,64]. These findings are consistent with the results obtained from the treatment of *V. pusilla* biomass with 70% and 75% H_3_PO_4_, and they reveal that these concentrations are incapable of decrystallizing cellulose. In addition, raising the CrI value of the pretreated biomass had no influence on the hydrolysis yields [10,19,48] since the highest rates of glucose recovery and digestibility were achieved after treatment with 75% H_3_PO_4_. The CrI value decreased when the sample was treated with 80% H_3_PO_4_ because the cellulose present in the treated biomass decomposes to a greater extent at higher concentrations. Moreover, during the regeneration of cellulose, the dissolved cellulose is reorganized, resulting in the modification and diminution of cellulose crystallinity [48,64,66,67]. In addition, at 85% H_3_PO_4_ concentration, the peak at 15.5° was missing, whereas the peak at 22.0° was displaced to a smaller angle, resulting in a broad asymmetrical peak with multiples at 12.0°, 19.9°, and 21.6° (Figure 3). At higher critical concentrations of H_3_PO_4_ (≥83%), high-order hydrogen bonds in the crystalline cellulose fibers were destroyed to a greater degree, resulting in cellulose I being converted into cellulose II. This enables the cellulose fiber to decompose entirely [42,49,68].

### 4.6. Bioethanol Fermentation

To ascertain the bioethanol production from the weed biomass, the *V. pusilla* BH produced by enzymatic hydrolysis was fermented using yeast. Several authors have reported that inhibitors are produced during the pretreatment of lignocellulose with acid, e.g., hydroxymethylfurfural (5-HMF), furfural, phenolics, and other aromatic compounds, which affect microbial fermentation [3,40,69]. Compared with the ST medium, the non-detoxified BH medium had a greater influence on yeast cell proliferation than on ethanol production, as the BH medium may include molecules that restrict the growth of yeast cells and ethanol production by *S. cerevisiae* TISTR 5339. Li et al. [70] demonstrated that a series of inhibitors, including vanillin, phenol, syringaldehyde, 5-HMF, furfural, levulinic acid, acetic acid, and formic acid, affect the development of *S. cerevisiae* during glucose fermentation. These findings suggest that the pretreatment of lignocellulosic materials with a high loading can result in higher sugar concentrations. However, this may also result in the development of a substantial number of inhibitors [24].

**Figure 6:**
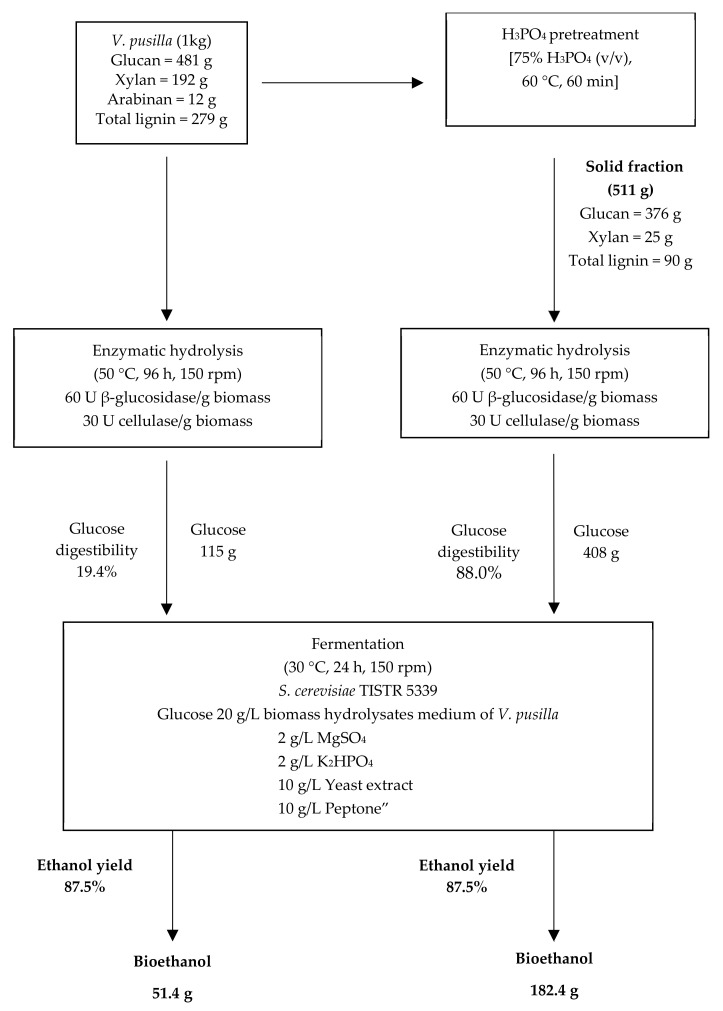
The mass balance of the *V. pusilla* feedstock for cellulosic ethanol production.

The ideal H_3_PO_4_ pretreatment conditions for fermentable sugar production were determined by calculating the mass balance of the *V. pusilla* biomass to assess the overall process (Figure 6). The main components of the feedstock were glucan (48.1%), xylan (19.2%), Arabinan (1.2%), AIL (23.5%), and ASL (4.4%). After pretreatment with 75% H_3_PO_4_, approximately 51.1% of the pretreated material was recovered, including 37.6% glucan, 2.5% xylan, 7.6% AIL, and 1.4% ASL. Both the untreated and pretreated *V. pusilla* biomass were hydrolyzed with cellulase (30 FPU/g substrate) and β-glucosidase (60 U/g substrate). After 96 h of hydrolysis, the untreated sample had a glucose digestibility of 19.4% and a recovery of 115 g/1 kg of biomass. However, the glucose digestibility and recovery of the treated *V. pusilla* biomass were enhanced to 88.0% and 408 g/1 kg of biomass, respectively. Compared with the untreated sample, glucose digestibility and recovery improved by approximately 4.5 and 3.5 times, respectively. Following the bioethanol production process, a non-detoxified BH medium containing 20 g glucose/L prepared from the treated *V. pusilla* biomass was fermented using *S. cerevisiae* TISTR 5339. As a result, approximately 51 g and 182 g of cellulosic ethanol were produced from the glucose in the untreated and pretreated *V. pusilla* biomass, respectively. Compared with the untreated sample, the bioethanol production of the treated *V. pusilla* biomass improved approximately 3.5-fold. Our study demonstrates that non-detoxified BH medium generated from *V. pusilla* feedstock can be used as the primary source of carbon for cellulosic ethanol production by *S. cerevisiae* TISTR 5339.

Overall, our findings provide crucial information and suggest that H_3_PO_4_ treatment of *V. pusilla* feedstock has the potential to enhance the production of bioethanol. However, we suggest utilizing the response surface methodology (RSM) as an experimental design in future studies to evaluate the impacts of significant pretreatment variables, including temperature, acid concentration, and duration, on solid recovery, glucose digestibility, and glucose recovery. To improve the ethanol yield by preventing inhibitory effects on organisms during fermentation, either the hydrolysate should be detoxified before being utilized as a substrate for bioethanol fermentation or organism-selected strains that are more resistant to inhibitors may be employed. This will streamline the process of producing cellulosic ethanol from weed biomass. However, microorganisms that can ferment the carbohydrates and micronutrients present in hydrolysate must be selected to obtain compounds with higher added value.

## 5. Conclusions

The pretreatment of *V. pusilla* biomass with varying concentrations of H_3_PO_4_ under mild conditions was more efficient in eliminating xylan than lignin. The optimum pretreatment condition for the greatest improvement in glucose recovery and digestibility was 75% H_3_PO_4_. Compared with the untreated sample, glucose digestibility and recovery improved by 4.5 and 3.5 times, respectively. The increase in both glucose recovery and digestibility yields may be attributable to the disintegration and dissolution of hemicellulose and lignin, which renders the cellulose present in the treated *V. pusilla* biomass vulnerable to cellulases. In addition, bioethanol fermentation in non-detoxified BH medium was established using treated *V. pusilla* biomass. Consequently, the ethanol yield was improved approximately 3.5-fold compared with that of the untreated sample. Our research revealed that sugar platform-based biorefineries are able to utilize this sort of *V. pusilla* biomass to produce biofuels and other valuable compounds.

## Figures and Tables

**Table 1:** Chemical composition of *V. pusilla*.

Composition (% DW)	*V. pusilla* (%)
Glucan	48.1 ± 0.3
Xylan	19.2 ± 0.4
Arabinan	1.2 ± 0.1
Ash	6.1 ± 0.1
Extractive	18.2 ± 0.2
AIL	23.5 ± 0.1
ASL	4.4 ± 0.1
Total lignin	27.9 ± 0.2

**Table 2:** Composition of *V. pusilla* after H_3_PO_4_ pretreatment.

Composition (% DW)	Raw Material	Concentration of H_3_PO_4_ (%)			
		70	75	80	85
Glucan	48.1 ± 0.3 ^c^	71.4 ± 0.3 ^b^	73.6 ± 1.1 ^a^	71.5 ± 0.7 ^b^	70.8 ± 0.8 ^b^
Xylan	19.2 ± 0.4 ^a^	7.6 ± 0.4 ^b^	4.8 ± 0.3 ^c^	4.0 ± 0.2 ^d^	n.d.
Arabinan	1.2 ± 0.1 ^a^	n.d.	n.d.	n.d.	n.d.
AIL	23.5 ± 0.1 ^a^	21.0 ± 0.6 ^b^	14.8 ± 0.7 ^c^	13.4 ± 0.3 ^d^	8.1 ± 0.7 ^e^
ASL	4.4 ± 0.1 ^a^	2.7 ± 0.1 ^b^	2.7 ± 0.0 ^b^	2.6 ± 0.0 ^b^	2.5 ± 0.0 ^c^
Total lignin	27.9 ± 0.2 ^a^	23.7 ± 0.6 ^b^	17.5 ± 0.7 ^c^	16.0 ± 0.3 ^d^	10.5 ± 0.7 ^e^
Solid recovery	100 ± 0.0 ^a^	53.8 ± 1.4 ^b^	51.1 ± 0.7 ^c^	50.2 ± 0.4 ^c^	46.8 ± 1.7 ^d^
Glucan recovery	100 ± 0.0 ^a^	79.9 ± 0.4 ^b^	78.3 ± 1.2 ^c^	74.7 ± 0.7 ^d^	68.9 ± 0.7 ^e^
Xylan recovery	100 ± 0.0 ^a^	21.4 ± 1.2 ^b^	12.8 ± 0.9 ^c^	10.4 ± 0.6 ^d^	n.d.
Arabinan recovery	100 ± 0.0 ^a^	n.d.	n.d.	n.d.	n.d.
AIL recovery	100 ± 0.0 ^a^	48.0 ± 1.4 ^b^	33.1 ± 0.2 ^c^	28.6 ± 0.6 ^d^	16.0 ± 1.5 ^e^
ASL recovery	100 ± 0.0 ^a^	32.7 ± 0.8 ^b^	30.6 ± 0.2 ^c^	29.9 ± 0.1 ^d^	26.0 ± 0.3 ^e^
Total lignin recovery	100 ± 0.0 ^a^	45.6 ± 1.1 ^d^	32.0 ± 1.2 ^c^	28.8 ± 0.5 ^d^	17.6 ± 1.2 ^e^
Total lignin removal	n.d.	54.4 ± 1.1 ^d^	68.0 ± 1.2 ^c^	71.2 ± 0.5 ^b^	82.4 ± 1.2 ^a^

The superscripted characters within the rows indicate the statistical significance of the differences between them (*p* < 0.05). n.d. = not determined.

**Table 3:** Crystallinity index (CrI) of untreated and H_3_PO_4_-pretreated *V. pusilla* samples.

	Raw Material	Concentration of H_3_PO_4_ (%)			
		70	75	80	85
**CrI (%)**	59.5	65.0	63.5	49.1	42.9

## Data Availability

Not applicable.
